# Pre-existing human rhinovirus infection modulates host response to secondary bacterial infections

**DOI:** 10.1186/1710-1492-10-S1-A57

**Published:** 2014-03-03

**Authors:** Jason Arnason, Kyla Jamieson, Cora Kooi, Sergei Nikitenko, Sami Shariff, Chris Shelfoon, David Proud, Richard Leigh

**Affiliations:** 1 Snyder Institute for Chronic Disease, University of Calgary, Calgary, Alberta, T2N 4N1, Canada

## Background

Secondary bacterial infections following viral infections of the airways are well documented and are associated with increased severity of respiratory disease compared to virus or bacterial infections alone. Human rhinovirus (HRV) infections are the most common causes of exacerbations in individuals with chronic airways diseases such as asthma and COPD. Moreover, bacterial colonization is commonly found in the airways of patients experiencing exacerbations of these chronic airways diseases and linked to increased severity and duration of these exacerbations. The mechanisms underlying the increased prevalence of secondary bacterial infections and the association with more severe outcomes following viral infections is not known. It has been suggested that viral infection of the airways cause dysregulation of innate host defense mechanisms, such as, impaired antimicrobial peptide expression of the airways. Antimicrobial peptides are key components of the innate immune response after infection and are important in efficient clearance of microbial colonization to prevent infection. We sought to determine whether HRV modulates the innate host defense response to secondary bacterial infections of the airways.

## Methods

Studies performed using primary human bronchial epithelial cells (HBECs). Cells grown in monolayer to confluence (80-90%). Antibiotics and Hydrocortisone were removed from the media 48 h and 24 h prior to infection respectively. On day of infection, cells were stimulated with purified HRV-16 or bacteria (*H. influenzae/P. aeruginosa*) alone, or treated in combination of HRV-16 then subsequently bacteria. TLR5 agonists (Flagella) also used alone and in combination with HRV-16. The protein and mRNA levels of different antimicrobial peptides (β-defensin, LL-37, lysozyme, lactoferrin, and SLPI) measured using ELISA (R&D Systems) and real-time RT-PCR (Applied Biosystems), respectively.

## Results

Preliminary data indicate supernatants from HRV-16/bacterial co-infection resulted in synergistic trend in β-defensin levels compared to HRV and bacteria alone. A synergistic increase in β-defensin levels was also seen with TLR5 agonist when combined post HRV-16 infection compared to TLR5 or HRV-16 alone. Minimum inhibitory concentration results showed β-defensin (1mg/ml), Lysozyme (30µg/ml), Lactoferrin (1mg/ml) and LL-37 (460µg/ml) were able to inhibit growth of NTHi and PAO.

## Conclusions

The data provide the first demonstration that there is a dysregulation of antimicrobial levels in HRV infected HBECs when encountered with a secondary bacterial infection. This provides evidence to why individuals with chronic airways diseases have a prolonged and more severe disease state than normal individuals. It could also lead to a new targeted therapy of people with diseases such as asthma or COPD to decrease severity of exacerbations.

